# Electrospun PVA-CTS-HA Wound Dressings with Ag-ZnO Nanoparticles for Diabetic Foot Ulcers Treatment: Physicochemical Properties, Hemocompatibility, and Cell Viability

**DOI:** 10.3390/polym17223001

**Published:** 2025-11-11

**Authors:** Karina Santiago-Castillo, Aidé Minerva Torres-Huerta, José. Manuel Cervantes-Uc, Adela Eugenia Rodríguez-Salazar, Silvia Beatriz Brachetti-Sibaja, Héctor Javier Dorantes-Rosales, Facundo Joaquín Márquez-Rocha, Miguel Antonio Domínguez-Crespo

**Affiliations:** 1 New Materials Department, Instituto Politécnico Nacional, CICATA-Altamira, Altamira, Altamira 89600, Tamaulipas, Mexico; k.sgcast@outlook.com; 2 Materials Department, Universidad del Noreste, Prol. Av. Hidalgo 6315, Tampico 89337, Tamaulipas, Mexico; 3 Nanostructured Materials Department, Instituto Politécnico Nacional, Unidad Profesional Interdisciplinaria de Ingeniería Hidalgo, San Agustín Tlaxiaca 42162, Hidalgo, Mexico; fmarquez@ipn.mx; 4 Biomaterials Department, Centro de Investigación Científica de Yucatán, Mérida 97205, Yucatán, Mexico; manceruc@cicy.mx; 5 Technological Innovation, Instituto Politécnico Nacional, CICATA Querétaro, Santiago de Querétaro 76090, Querétaro, Mexico; aerodriguez@ipn.mx; 6 Tecnológico Nacional de México, Instituto Tecnológico de Cd. Madero, Ciudad Madero 89440, Tamaulipas, Mexico; silvia.bs@cdmadero.tecnm.mx; 7 Metallurgical Department, Instituto Politécnico Nacional, Escuela Superior de Ingeniería Química e Indusrtrias Extractivas, Mexico City 07738, Mexico; hdorantes@ipn.mx

**Keywords:** PVA, chitosan, Hyaluronic acid, inorganic nanoparticles, scaffolds, diabetic foot ulcer

## Abstract

Diabetic foot ulcers (DFUs) are complex to heal and can lead to amputations and high healthcare costs. To address this, a promising alternative is the creation of electrospun fiber scaffolds for wound dressings. This study fabricated these scaffolds using a blend of natural polymers—chitosan (CTS), polyvinyl alcohol (PVA), and hyaluronic acid (HA)—along with antibacterial silver (Ag) and zinc oxide (ZnO) nanoparticles. The researchers conducted comprehensive analyses, including physicochemical, morphological, and biological assessments. The Ag structures showed potential as microbicidal agent, while the ZnO nanoparticles demonstrated photoactivity and the ability to generate reactive oxygen species (ROS) for antibacterial action. The resulting PVA-CTS-HA-Ag-ZnO scaffolds were found to be both hemocompatible and non-hemolytic, meaning they are safe for use with blood. The cytotoxicity evaluation using the ISO 10993-5 standard showed that the incorporation of CTS and HA decreased cytotoxicity of pure PVA, obtaining non-cytotoxic scaffolds (viability > 70%). Electrospun scaffolds composed with Ag-ZnO NPs in 50-50 and 70-30 ratios also maintained this biocompatibility, while the 30-70 ratio (Ag-ZnO) showed a cytotoxic effect, suggesting a ZnO concentration-dependent effect. These findings confirm that these materials are suitable for supporting skin cell regeneration, having a high potential for use as interactive dressings for treating chronic wounds.

## 1. Introduction

Diabetic foot ulcers (DFUs) are a prevalent and severe complication in patients with diabetes mellitus. Individuals with diabetes often experience anatomical and functional changes in the lower limb, mainly as a result of diabetic peripheral neuropathy, peripheral vascular disease, or both. [1,2]. These changes significantly heighten susceptibility to infection, ulceration, and deep tissue destruction, leading to high rates of disability and mortality [3]. The global burden of diabetes is substantial and growing. According to the International Diabetes Federation (IDF), there were an estimated 537 million people living with diabetes worldwide in 2021, with a projection of 783 million by 2045 [4,5,6]. In this context, it is estimated that one amputation occurs every 20 s globally due to diabetes-related complications [7], with DFUs being the most frequent chronic complication associated with the disease [8]. Furthermore, of these, around 15–25% are expected to develop a DFU, and around 50% of these individuals will experience a recurrence within a short period, significantly impacting their personal lives and work productivity [4]. The economic strain of DFUs on healthcare systems is considerable, with annual costs ranging from $1892 to $13,595 per patient. In the USA, for example, the direct cost of diabetes treatment was estimated at $237 billion in 2017, with one-third of this amount allocated to the care of diabetic foot disease. Ultimately, 17% of these patients require amputation [9,10]. A striking fact is that 80% of these amputations could be prevented through adequate treatment and the implementation of robust prevention measures [11].

Chronic hyperglycemia interferes with almost every stage of the healing process, making wound healing in diabetic patients a complex and deficient process compared to non-diabetic patients. Hyperglycemia damages nerves and blood vessels, causing neuropathy and atherosclerosis, as well as affecting the functioning of endothelial cells, which are essential for wound healing [12]. Neuropathy occurs when glucose damages the peripheral nerves, causing a lack of protective sensitivity in the feet that increases the risk of developing trauma and ulcers [13,14]. Atherosclerosis occurs when hyperglycemia causes hardening of the arteries, which reduces blood flow to the extremities, preventing the wound from receiving oxygen and nutrients [15]. Another effect of hyperglycemia is to alter the migration and proliferation of keratinocytes and fibroblasts, which are essential for the re-epithelialization process [16]. For patients with diabetes who have developed complications, the formation of Advanced Glycation End Products (AGEs) has been associated with structural changes in key skin proteins, such as collagen and elastin, resulting in a weak skin structure [17]. In addition, because the immune system is weakened, the wound can become infected more easily [18].

Treatments for DFUs are tailored to the severity of the lesions and typically involve advanced dressings, antibiotic therapy, debridement of necrotic tissue, and surgical revascularization techniques. Amputation is considered when ischemia or gangrene is present [12]. Unfortunately, 40–70% of patients with chronic ulcers fail to heal due to impaired healing capacity, underscoring the critical need for innovative therapeutic approaches.

An ideal material for advanced wound dressings, must possess specific characteristics to prevent infection and accelerate healing. These include being non-toxic and highly biocompatible with the cells of the affected tissue, maintaining a moist wound environment, allowing gas exchange, effectively absorbing exudates, and providing a robust barrier against microbial penetration (e.g., *Pseudomonas aeruginosa*, *Escherichia coli*, *Staphylococcus aureus*, *Staphylococcus epidermidis*, and *Enterobacter* species) [19]. Additionally, the dressing must exhibit adequate mechanical properties to protect the wound from external forces, promote fibroblast proliferation, and facilitate re-epithelialization, thereby enhancing wound healing. Finally, the dressing material should be cost-effective and cause minimal discomfort to the patient [20]. A wide range of wound dressings—including sponges, hydrogels, foams, and films—have been developed using various fabrication techniques [21,22,23,24,25].

In recent years, significant research has focused on fabricating extracellular matrix-like scaffolds from electrospun fibers for wound dressing applications [26]. Electrospinning is an electrohydrodynamic technique used to produce micro- or nanometer-range fibers from polymeric solutions or molten polymers, utilizing natural, synthetic, or blended materials [27]. Generally, natural polymers offer superior biocompatibility and biodegradability, while synthetic polymers are easier to process into electrospun fibers and exhibit better mechanical properties [28]. More recently, the incorporation of bioactive molecules, organic, or inorganic nanostructures has been explored to enhance the properties of these polymers [29,30,31,32].

The use of chitosan (CTS) combined with other compounds like polyvinyl alcohol (PVA), nanocellulose, hyaluronic acid (HA), biocompatible materials, and antibiotics to produce electrospun fibers has emerged as a promising option for DFU treatment. This promise stems from their synergistic properties that promote cell adhesion and proliferation, alongside characteristics such as high porosity and low toxicity [33,34,35,36]. CTS, a natural polymer, is biocompatible, biodegradable, hemostatic, antibacterial, and antifungal, and actively promotes cell proliferation and wound healing [37]. Furthermore, CTS exhibits strong antioxidant activity, making it an excellent material for tissue engineering, controlled pharmaceutical delivery, and wound dressings [38]. PVA is a water-soluble, biodegradable, and biocompatible synthetic polymer that exhibits enhanced thermal and chemical stability, resulting in low protein adsorption efficiency for bio-adhesive applications [39]. PVA is compatible with many polymers and is frequently incorporated into CTS to enhance the uniformity, and the mechanical, biodegradable, and hydrophilic properties of CTS nanofiber scaffolds [40,41]. The incorporation of PVA into CTS also enhances the biocompatibility of electrospun fiber scaffolds by promoting fibroblast viability, proliferation, and gene expression [42].

Hyaluronic acid (HA) possesses crucial properties for its application in wound and burn healing dressings, primarily by promoting cell proliferation, migration, and wound healing. Its specific features include inducing keratinocyte differentiation, proliferation, and adhesion, as well as stimulating fibroblast proliferation and gene expression in macrophages, endothelial and epithelial cells, and eosinophils. During the early stages of wound healing, HA plays a crucial role in forming a temporary structure at the wound site, which is essential for nutrition, cleansing, and the addition of proteoglycans and collagen [43]. However, using HA to prepare electrospun fibers for biomedical applications remains a significant challenge. This is due to its high-water solubility and polyanionic nature, which results from the carboxylic groups (-COOH) in its structure. These lead to long-range electrostatic interactions and the presence of counterions, which increase viscosity but prevent polymer chain entanglements, thereby hindering its processability by electrospinning [44].

Another major limitation in the electrospinning production of HA mats for dressing materials is their poor mechanical properties. Recent reports have highlighted the preparation of composites based on CTS-HA blends with other polymers or chemical compounds to improve overall mechanical properties or biocompatibility [45,46]. For example, blends of PVA-CTS-HA with inorganic nanoparticles have been reported for electrospun fiber dressing material applications [47,48,49,50]. Most researchers have found that PVA in these blends acts as a physical barrier, preventing electrostatic interactions. Meanwhile, CTS and HA, owing to their cationic and anionic natures, respectively, exhibit mutual attraction. This suggests that HA can function as a binding agent for CTS, improving its structural stability and prolonging its lifetime during the healing process [51]. It has also been observed that the use of silver (Ag) or zinc oxide (ZnO) nanoparticles (NPs) in PVA-CTS-HA-based dressings improves their antibacterial activity and offers the benefits of nanoscale materials, such as high reactivity and surface area [49,52]. A significant advantage of these inorganic NPs is that pathogenic bacteria are less likely to develop resistance to them [53,54]. Furthermore, they exhibit low toxicity and enhance collagen deposition at the wound site, thereby promoting wound healing [55]. Specifically, silver NPs, can accelerate wound healing by stimulating keratinocyte proliferation and migration, regulating cytokine release, and decreasing wound inflammation [56].

The goal of this work is to fabricate biodegradable, biocompatible, and non-cytotoxic scaffolds from electrospun fibers by optimizing the PVA-CTS-HA ratio. Furthermore, this study aims to analyze the effect on the antibacterial and anti-inflammatory properties when 1.95 g of CTS-Ag-ZnO NPs, at different ratios (30, 50, and 70 wt.%), are incorporated into these electrospun composites. The biological analysis will include the evaluation of cell viability and hemocompatibility of the developed materials. This work hypothesizes that optimizing the PVA-CTS-HA inorganic nanoparticle ratio and the synthesis parameters in the course of the electrospinning will elucidate the synergistic effect of the components on the physicochemical and biological properties, thereby fostering the desired characteristics in dressing materials for DFU treatment applications. The importance of this research lies in the urgent need to find effective alternatives for DFU treatment, which not only reduces the amputation rate but also greatly enhances the overall well-being of individuals with diabetes. By developing and optimizing this proposal, we aim to contribute to innovation in the field of regenerative medicine and chronic wound treatment, thus providing a viable solution to a public health problem of growing relevance.

## 2. Materials and Methods

### 2.1. Raw Materials

The experimental study involved the synthesis of nanocomposites and electrospun fibers using a variety of reagents. Chitosan (CTS), sourced from Sigma-Aldrich (Naucalpan de Juárez, Mexico), had an average molecular weight of 190–310 kDa, a 75–85% degree of deacetylation, and 90% purity. Silver nitrate (AgNO_3_) (Sigma Aldrich, 99% purity) and D-(+)-glucose (C_6_H_12_O_6_) (Sigma Aldrich, 99% purity) were used. Glacial acetic acid (C_2_O_2_H_4_) from Fermont (San Luis Potosí, Mexico) had a purity of 99.8%. Zinc acetate dihydrate (C_4_H_6_O_4_Zn·2 H_2_O) (Sigma Aldrich, 98% purity) and sodium hydroxide (NaOH) (Fermont, 98.7% purity) were also utilized. Polyvinyl alcohol (PVA) (Sigma Aldrich) had a molecular weight range of 146–186 kDa, was >99% hydrolyzed, and had 99.3% purity. Hyaluronic acid (HA), with a molecular weight of 1.3–1.6 MDa and 99% purity, completed the primary list of reagents for material synthesis. All reagents were used without further purification.

For the cell culture assays, Detroit 548 fibroblasts were generously provided by the Institute of Biotechnology of the Autonomous University of Nuevo Leon. Additional reagents included physiological saline solution (PISA, Monterrey, Mexico), Wright’s staining kit (HYCEL, Zapopan, Mexico), and collagen fibrogel (Sigma Aldrich). Cell culture media and supplements consisted of Dulbecco’s Modified Eagle’s Medium (DMEM, NY, USA), fetal bovine serum (FBS), a penicillin/streptomycin antibiotic and antifungal solution, and 0.25% trypsin-EDTA solution (Gibco, Sigma Aldrich, Mexico City, Mexico).

### 2.2. Methodology

The synthesis of electrospun PVA-CTS, PVA-CTS-HA, PVA-CTS-HA-Ag-ZnO NPs fibers was carried out based on previous reports [57,58]. To produce the PVA-CTS electrospun fibers, the starting point was the conditions of previous work [59], which included a PVA-CTS ratio of 60–40 wt% and varied the process parameters. After morphological optimization of the PVA-CTS-HA fibers, the composite fibers, were fabricated with the weight ratios shown in Table 1. Each sample was processed by electrospinning for 30 h.

#### 2.2.1. Physicochemical Characterization of the Electrospun Scaffolds

The analysis of the powders and scaffolds by Fourier Transform Infrared Spectroscopy (FTIR) was carried out without any prior sample preparation, utilizing the attenuated total reflectance (ATR) mode (ZnGe) and an air blank. The equipment used was a Perkin Elmer, Spectrum One model (Mexico City, Mexico). Sample measurements were performed using a frequency range of 4000–650 cm^−1^ at a resolution of 4 cm^−1^. Sixteen scans were performed per sample.

The structural analysis of powder and scaffold samples was performed using a Bruker D8 Advance diffractometer (Mexico City, Mexico), which operates with a Bragg-Bretano geometry (θ–2θ), voltage of 40 kV, a current of 40 μA and Kα radiation of Cu (λ = 1.5406 Å). The XRD pattern was recorded in the range of 5° to 100° degrees 2θ. The powder samples were placed in the corresponding sample holder without any prior preparation, while the CTS- Ag NPs samples and scaffolds were analyzed as films.

To verify the synthesis of Ag and ZnO nanoparticles in the chitosan matrix, UV-Vis analysis was performed. For this analysis an Agilent Technologies spectrophotometer, Cary series 5000 (Mexico City, Mexico), was used in absorbance mode for the CTS-Ag NPs compound (kept at constant volume) and in diffuse reflectance mode for the CTS-ZnO NPs compound. The analysis was performed in a wavelength range of 200–800 nm.

The morphological analysis of the CTS-Ag and CTS-ZnO composites was carried out using Scanning Electron Microscopy (SEM). The morphology of both powders and scaffolds was analyzed using JEOL JSM-6701F High Resolution Electron Microscopy (Mexico City, Mexico). To perform a semiquantitative analysis of the elements present in the samples, an **E**nergy **D**ispersive **S**pectroscopy (EDS) analysis was carried out, which was coupled to SEM. Sample preparation consisted of depositing a gold/palladium coating on the powder or fiber mat samples (attached to a conductive carbon ribbon), using an Emitech SC7640 cathodic coating device (Sussex, UK), for 90 s.

The thickness of the electrospun scaffolds was determined with a Mitutoyo digital micrometer (Mexico City, Mexico), with a measuring range of 0 to 1” with a resolution of 0.00005” and an accuracy of ±0.00005”.

#### 2.2.2. Biological Assessment

The percentage of hemolysis was determined following the ISO 10993-4 standard for the evaluation of the biocompatibility of medical devices. A blood sample was obtained from a healthy individual and collected in a plain tube, which is a whole blood tube without an anticoagulant. This assay was performed within 4 h of sample collection. The procedure consisted of placing scaffold samples with a disc morphology, 3 mm in diameter in contact with 0.6 mL of 5% erythrocyte solution. Previously, the scaffold samples were sterilized by UV radiation in a laminar flow hood for 30 min per side. Afterwards, the scaffold samples with blood cells were centrifuged for 10 min, at 1500 rpm, 20 °C. 10 µL of supernatant was taken and diluted with 0.85% physiological saline. Absorbance was recorded at λ = 415 nm in a multimodal plate reader (Cytation 3, Biotek, Mexico City, Mexico) to determine hemoglobin release caused by hemolysis. Each experiment was conducted in triplicate. Subsequently, the percentage of hemolysis was calculated for each sample according to Equation (1):

(1) $$Hemolysis\;(\%)=\frac{A_{sample}-A_{negative\;\_control}}{A_{positive\_control}-A_{negative\_control}}\times 100$$

In the equation: A sample refers to the absorbance of the sample, while A negative_control denotes the absorbance of the negative control (blood with physiological saline or a non-hemolytic material) and A positive_control represents the absorbance of the positive control (blood with distillated water to induce 100% hemolysis).

A viability assay was performed by direct contact with the cells to evaluate their adhesion to the scaffolds. Because the scaffolds floated in the medium, poly(lactic acid) (PLA) rings were made to hold them at the bottom of the culture wells. For this, PLA rings with an external diameter of 6 mm and an internal diameter of 4 mm were 3D printed. These rings were washed with 2% Dextran^®^ and sterilized in ethanol for 24 h. Scaffold discs of 4 mm diameter were prepared and placed in a 96-well plate. The substrates were sterilized for 30 min by UV irradiation and washed twice with sterile PBS. Subsequently, cells were seeded on the substrates at a density of 5 × 10^3^ cells per well and incubated with 100 µL culture medium for 24 h.

**Figure 1:**
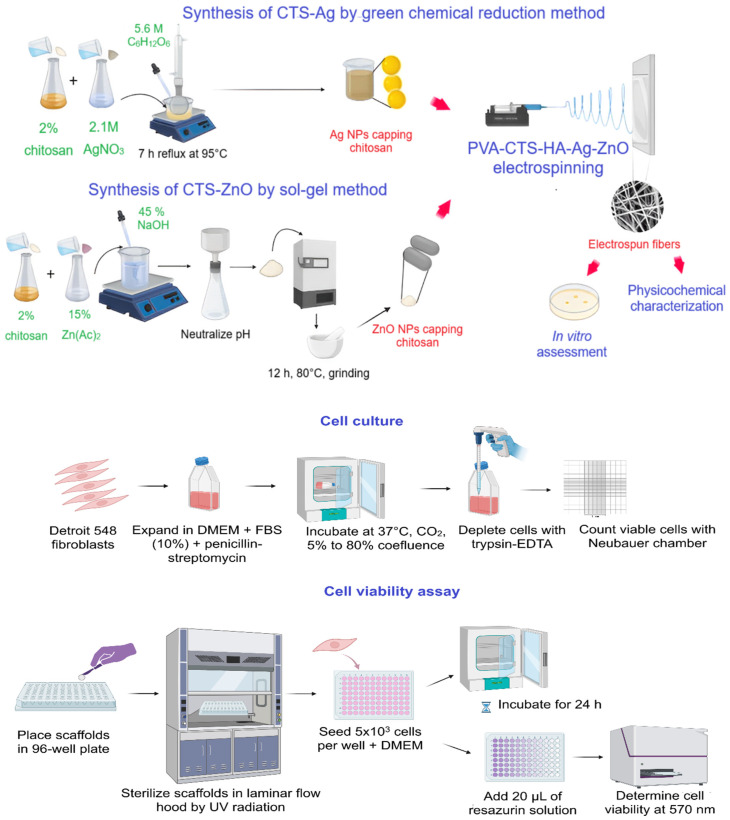
Experimental setup of PVA-CTS-HA-Ag/ZnO electrospun fiber scaffolds and their evaluation.

After this, cells were observed under a Labomed TCM 400 inverted optical microscope. To evaluate cell viability, 20 µL of resazurin solution was added to the wells and incubated for 4 h. Subsequently, the absorbance of the wells was measured at 570 nm in a BioTek Cytation 3 plate reader, Mexico City, Mexico. A well without scaffolds and with the same number of cells was used as a reference control (representing 100% viability). Cell viability was reported as the average of 5 measurements using the following equation:

(2) $$Cell\;viability\;(\%)=\frac{A-A_{n}}{A_{p}-A_{n}}\times 100$$

where A is the average of the absorbance of the wells of each treatment, A_p_ represents the absorbance of the positive control and A_n_ represents the absorbance of the negative control; see Figure 1 for experimental setup of PVA-CTS-HA-Ag/ZnO electrospun fiber scaffolds and their evaluation.

## 3. Results and Discussion

### 3.1. Chemical Characterization of Binary CTS-Ag and CTS-ZnO Compounds

**Figure 2:**
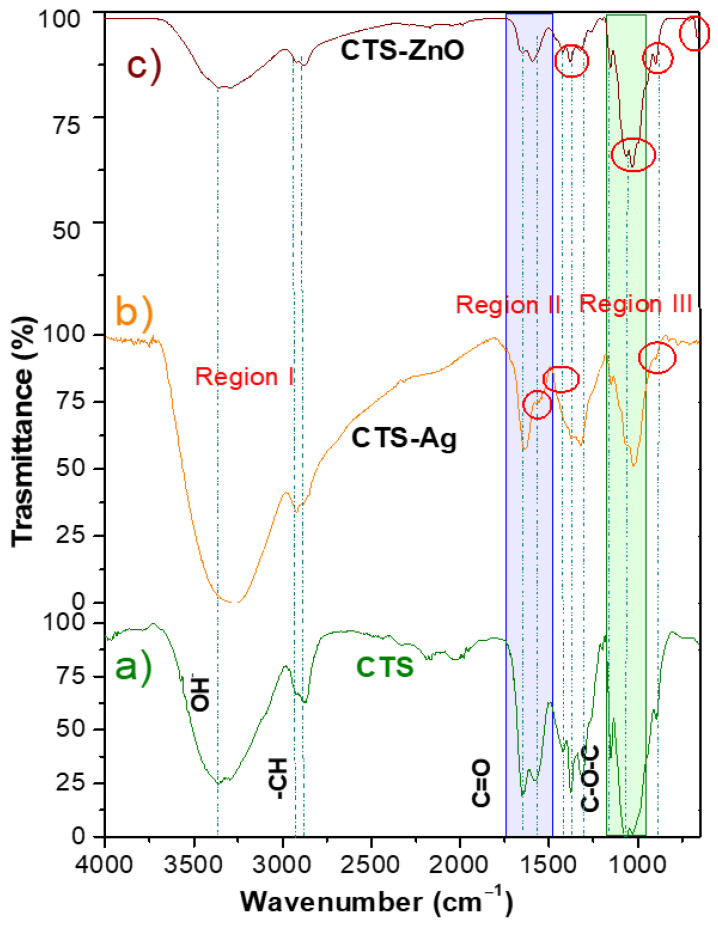
FT-IR transmittance spectra of (**a**) CTS pure, (**b**) CTS-Ag and (**c**) CTS-ZnO compounds.

Figure 2 presents the FTIR spectra of the CTS-Ag and CTS-ZnO compounds and compares them with the spectrum of pure CTS to identify changes in absorption bands that confirm the formation of silver assemblies. CTS is a linear polysaccharide composed of D-glucosamine units linked via glycosidic (C-O-C) bonds and is rich in hydroxyl and amino groups. Particularly, in the FTIR spectra of pure CTS (Figure 2a), the characteristic bands of these groups can be highlighted: the overlap of the -OH and -NH_2_ amino bands at the wavenumber range of 3375–2976 cm^−1^ [60], the secondary stretching of -OH groups is identified at 1376 cm^−1^, while the asymmetric stretching of -CH groups can be detected at 2922 and 2872 cm^−1^. [61]. Furthermore, the typical bands for carbonyl (C=O) stretching of amides and amide bands can be observed at 1648 cm^−1^ and 1567 cm^−1^, respectively. Finally, the C-N stretching of amide III can be observed at 1315 cm^−1^, while the band at 1065 cm^−1^ corresponds to the stretching of the glycosidic linkage [62]. For comparison, the CTS spectrum can be divided into three regions: (I) the superimposed vibration bands characteristic of the -OH and -NH_2_ functional groups, located between 3400–2500 cm^−1^; (II) the region between 1700–1200 cm^−1^, which is characteristic of amide groups; and (III) the region between 1200–800 cm^−1^, characteristic of the saccharide structure [63].

In the spectrum of the CTS-Ag compound, shifts were observed towards both higher and lower wavenumbers compared to the pure CTS spectrum in regions I and II (Figure 2b). For example, the band corresponding to the superposition of the -OH and -NH_2_ functional groups, which in the CTS spectrum appears at 3362 cm^−1^, shifted to lower wavenumbers in the CTS-Ag compound (3278 cm^−1^), while the bands corresponding to -CH, which appear between 2922 and 2872 cm^−1^ in CTS, shifted to higher wavenumbers (2919 cm^−1^) in the composite sample, and the intensity of the band also increased. The observed changes in the FTIR spectrum of CTS-Ag are attributed to the physical interactions between silver nanoparticles and CTS. According to Berthomieu et al., a decrease in the stretching vibration frequency is related to an increase in the mass of the atoms forming the group [64]. This indicates that shifts to lower wavenumbers are due to the interaction of the -OH and -NH_2_ functional groups of CTS with silver nanostructures, increasing the effective mass and, consequently, reducing the vibration frequency. On the other hand, shifts to higher wavenumbers (increase in vibration frequency) can be related to a decrease in bond length or the inhibition of hydrogen bonds [65]. This is the case for the C-H linkage, which in pure CTS appears at 2869 cm^−1^. In the CTS-Ag compound, the disappearance of some bands in region II related to amide groups of CTS was observed. The bands at 1572 cm^−1^ (N-H bending of amide), and 1424 cm^−1^ 0 (-CH_2_ bending) disappeared, suggesting that the remaining amide groups from the deacetylation of CTS interact with the Ag particles. Changes in intensity were also observed in the CTS absorption bands at 1375, 1306 and 1151 cm^−1^, corresponding to the stretching of the -OH group, C-N stretching of amide II, and the C-O-C linkage, respectively. In region III, a decrease in the intensity of the bands at 1075 cm^−1^ (C-O stretching) and 886 cm^−1^ (out-of-plane CH bending of monosaccharides), was observed [66]. This decrease can be attributed to the fact that the molecules are not in a pure state but are in a mixture and, therefore, at different concentrations.

The CTS-ZnO spectrum also shows features similar to CTS, with some key differences (Figure 2). For example, these signals are evident in the CTS absorption bands, including the band attributed to stretching vibrations of the -OH and -NH_2_ groups appears more reduced, which can be attributed to the formation of inter- and intramolecular bonds in the presence of the ZnO particles [58]. The main changes in the chemical environment, attributed to the synthesis of the ZnO particles, were reflected in the intensity of the bands at 1572 and 1031 cm^−1^, which correspond to the N-H bending of amide II and the C-O-C stretching, respectively. Additionally, the band at 896 cm^−1^, corresponding to the out-of-plane -CH bending of the monosaccharide ring, overlaps with a new band at 876 cm^−1^, attributed to the Zn-O interaction.

### 3.2. UV-Vis Spectroscopy of CTS-Ag and CTS ZnO Compounds

**Figure 3:**
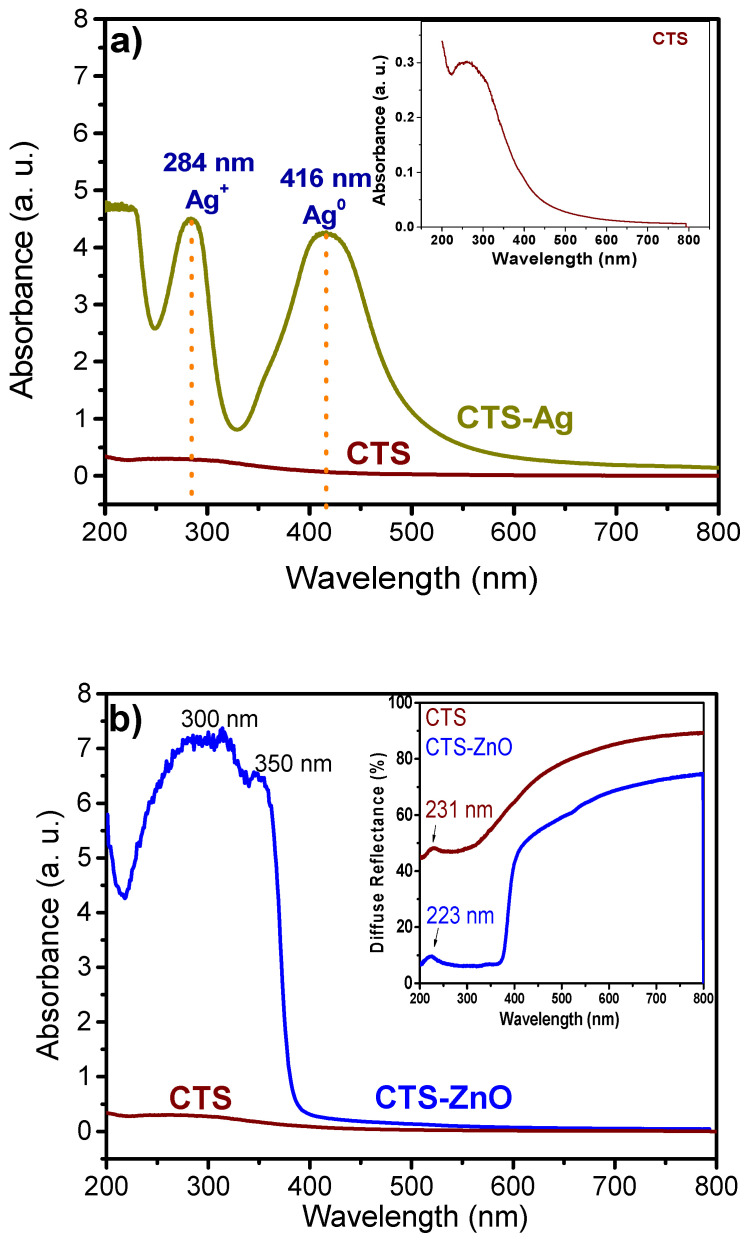
UV-Vis of the absorption spectra of (**a**) CTS-Ag and (**b**) CTS-ZnO compounds and its comparison with pure chitosan. In (**b**) it is also shown the diffuse reflectance of the CTS-ZnO compounds.

Metallic particles have a high capacity for light absorption, leading to the phenomenon of surface plasmon resonance (SPR). This phenomenon, crucial for their identification, occurs when the conduction electrons of metallic particles are excited by light at a specific wavelength, generating a collective oscillation [67]. Figure 3a presents the UV-Vis spectrum of the CTS-Ag compounds, evaluated in absorbance mode to observe the formation of silver particles in the CTS matrix. The spectrum displays two absorption signals: one at 416 nm and another at 284 nm. The 416 nm band is attributed to basal silver (Ag^0^) and corresponds to the presence of the SPR band, while the 284 nm signal is associated with cationic silver (Ag^+^). Similarly to previous reports on CTS-Ag compounds, the high absorbance values and well-defined band suggest the presence of basal silver in high concentrations [57,68]. The optical properties of Ag particles are intrinsically linked to their size, shape, and aggregation state. In this context, Paramelle et al., demonstrated that 20 nm silver nanospheres exhibit the SPR effect near 400 nm, while larger sizes cause broadening and a redshift (longer wavelengths) of the absorption signal [69]. Likewise, particle aggregation causes a redshift of the SPR signal, since conduction electrons at the surface are shared among neighboring particles, reducing the energy required for the phenomenon [70]. Based on these findings, the size of the Ag particles synthesized in this study appears to be close to that reported by Abdelgawad et al., around 20 nm [57]. This suggests that the formation of nanometric Ag particles within the CTS matrix was effective.

Quantum confinement can significantly alter the optical properties of semiconductor materials like ZnO and TiO_2_. For this reason, the CTS-ZnO compounds were analyzed using UV-Vis spectroscopy to observe electronic transitions of the materials and identify possible shifts in absorption bands attributable to the ZnO particle size.

Figure 3b shows the UV-Vis spectrum in absorbance and diffuse reflectance modes of pure CTS (wine color) compared with the CTS-ZnO compounds (blue line). In diffuse reflectance, the CTS spectrum exhibits a weak signal at 231 nm, attributed to the n→π transition of the amide groups [71]. In the CTS-ZnO compound spectrum, this signal shifts slightly to a shorter wavelength (223 nm). This blueshift (shorter wavelengths) can be attributed to the quantum effects of the ZnO particles, due to their interactions with CTS.

It has been reported that ZnO nanostructures exhibit electronic transitions in the visible light range, from 350–375 nm [72], leading to the appearance of absorption bands in this region. The band at 350 nm corresponds to the ZnO NPs. As expected, the spectrum of the CTS-ZnO compound shows higher absorbance percentages in the UV region (220–370 nm) than pure CTS, which aligns with the results reported by Ocakoglu et al. [73]. The main recognized antibacterial action of ZnO nanoparticles arises from the generation of reactive oxygen species (ROS) through photocatalysis in an aqueous medium. Therefore, the band gap energy (Eg) of the CTS-ZnO compound was determined. To achieve this, the Kubelka-Munk function F(R∞) was utilized to convert the diffuse reflectance spectra into equivalent absorption spectra. The band gap was then determined from the slope of the linear region in the (F(R∞)hv)^2^ versus hv (photon energy) plot.

The function was extrapolated, and a value of 3.25 eV was determined as E_g_, equivalent to 381.5 nm. This value coincides with what has been reported for ZnO nanostructures synthesized by sol–gel [72]. Contrary to what was expected, no blueshift caused by the quantum effect was obtained. Nevertheless, the CTS-ZnO compound showed a decrease in the band gap compared to the bulk material (3.37 eV). This can be attributed to the presence of chemical defects or vacancies in the intergranular regions, which generate a new energy level that reduces the optical band gap [73].

### 3.3. XRD of CTS-Ag and CTS-ZnO Compounds

**Figure 4:**
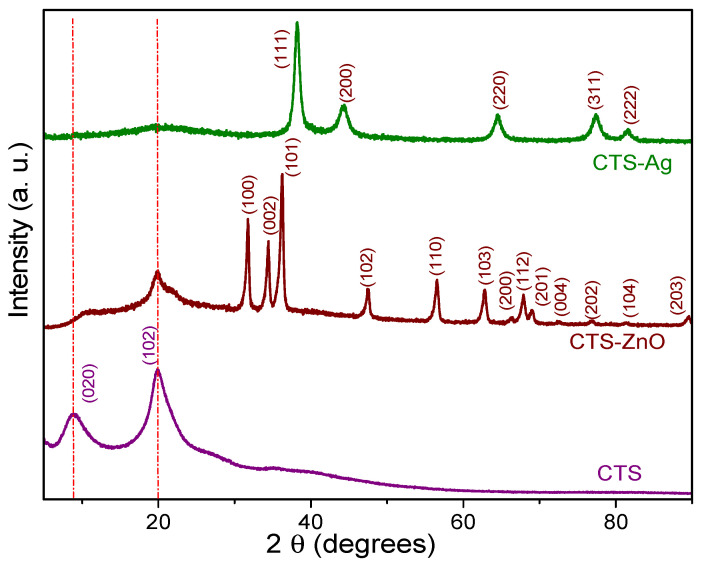
XRD patterns of pure CTS, CTS-Ag and CTS-ZnO compounds.

Figure 4 illustrates the structural changes that occur when inorganic nanoparticles (Ag and ZnO) are added to the Chitosan (CTS) matrix, compared to pure CTS. The diffractogram of pure CTS shows two distinct reflections at 2θ = 9.02° and 20.08°, which are assigned to the α and γ phases, respectively (PDF #00-039-1897). These reflections indicate the semicrystalline nature of CTS, attributed to intermolecular and intramolecular hydrogen bonding interactions that impart regularity to its structure [74]. In contrast, in the diffractogram of the CTS-Ag composite, marked alterations were observed in the characteristic reflections of CTS. For example, the reflection at 9.02° (α phase) completely disappeared, while the reflection at 20.08° (γ phase) shifted to 19.6° and showed a substantial reduction in intensity. These changes suggest a loss of the polymer’s semi-crystalline nature in the presence of Ag structures, likely due to a reduction in hydrogen bonds [75].

Additionally, the CTS-Ag diffractogram presents characteristic reflections of cubic silver, located at 2θ ≅ 38°, 44°, 64°, 77°, and 81°. These reflections correspond to the crystallographic planes (111), (200), (220), (311), and (222), respectively, and match the diffraction pattern of crystallographic card PDF #01-087-0717 [76]. In the case of the CTS-ZnO compounds, a reduction in the intensity of the signals corresponding to the (020) and (102) planes of CTS was observed. This reduction is attributed to the synthesis of ZnO structures, possibly due to the interaction between the acetyl groups of zinc acetate and available NH_2_ molecules to form hydrogen bonds [75]. Notably, the signal of the (102) plane of CTS in the CTS-ZnO composite showed a shoulder (19.98°), due to the interaction with the inorganic particles. Additionally, the crystalline signals of ZnO NPs can be observed. The distinctive peaks of the hexagonal structure of ZnO appears at 31.9° (100), 34.6° (002), 36.4° (101), 47.5° (102), 56.6° (110), 62.90° (103), 66.5° (200), 68.0° (112), 69.17° (201), 72.5° (004), 77.0° (202), 81.21° (104), and 89.35° (203), which match well with the PDF #00-036-1451. An estimation of the crystallite size was carried out using the Scherrer equation, $d(nm)=\frac{0.9\lambda }{\beta cos\theta }$, where d is the crystallite size, λ is the wavelength of the incident radiation, β is the FWHM (Full Width at Half Maximum) of the reflection and θ is the angle of the reflection in radians. The average crystallite size (C.S.) of the Ag structures in the composite was 9.27 ± 0.52 nm, for ZnO, it was. 17.77 ± 1.71. As a consequence of the addition of inorganic nanostructures and their interaction with the polymer matrix, the C.S of CTS slightly increased from 2.94 ± 0.10 nm to 3.99 ± 1.47 nm.

### 3.4. Morphological and Microstructural Observations

**Figure 5:**
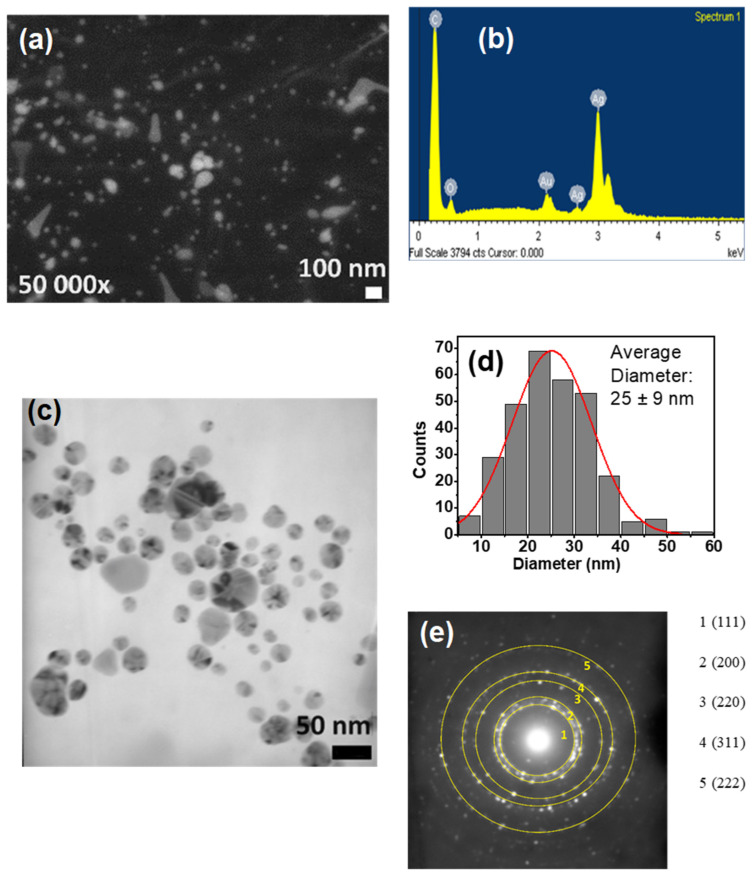
Morphological analysis of the CTS-Ag compound: (**a**) SEM image of the CTS-Ag compound at 50,000×, (**b**) EDS diagram confirming the presence of Ag, (**c**) TEM image at a magnification of 100,000×, (**d**) average diameter distribution of inorganic nanoparticles and (**e**) Selected Area Electron Diffraction (SAED) pattern showing the planes corresponding to silver structure. In agreement with these studies, the CTS acts as a stabilizer, reducing the particle size of the ZnO NPs [78,79]. The Transmission Electron Microscopy (TEM) images show a hexagonal shape that matches well with the Debye-Scherrer rings observed by selected-area electron diffraction (SAED) for (100), (002), (101), (102), and (110), with a particle size that agrees with the X-ray diffraction (XRD) analysis (Figure 6e).

Figure 5a–e provides a detailed morphological and microstructural analysis of the CTS-Ag composite. Scanning Electron Microscopy (SEM) images (Figure 5a) show that silver (Ag) structures are deposited on the surface of the CTS matrix, displaying an irregular shape and a wide size distribution. This was verified by Energy-Dispersive X-ray Spectroscopy (EDS) (Figure 5b), which detected silver along with carbon and oxygen from the polymeric matrix. These findings collectively confirm the successful formation of Ag nanostructures within the CTS matrix, which is consistent with previous studies, such as that of Badawy et al. [77]. Transmission Electron Microscopy (TEM) images (Figure 5c) offered a closer look, revealing that the silver particles have a spherical morphology with an average diameter of 25 ± 9 nm, a measurement that aligns with estimates from UV-Vis spectroscopy. The Selected Area Electron Diffraction (SAED) pattern (Figure 5e) provided additional structural support. The calculated interplanar distances correspond to the (111), (200), (220), (311), and (222) planes, which are characteristic of a cubic silver structure (PDF # 01-087-0717). This finding also confirms prior estimations from X-ray diffraction (XRD), providing robust validation of the composite’s successful synthesis and structure.

**Figure 6:**
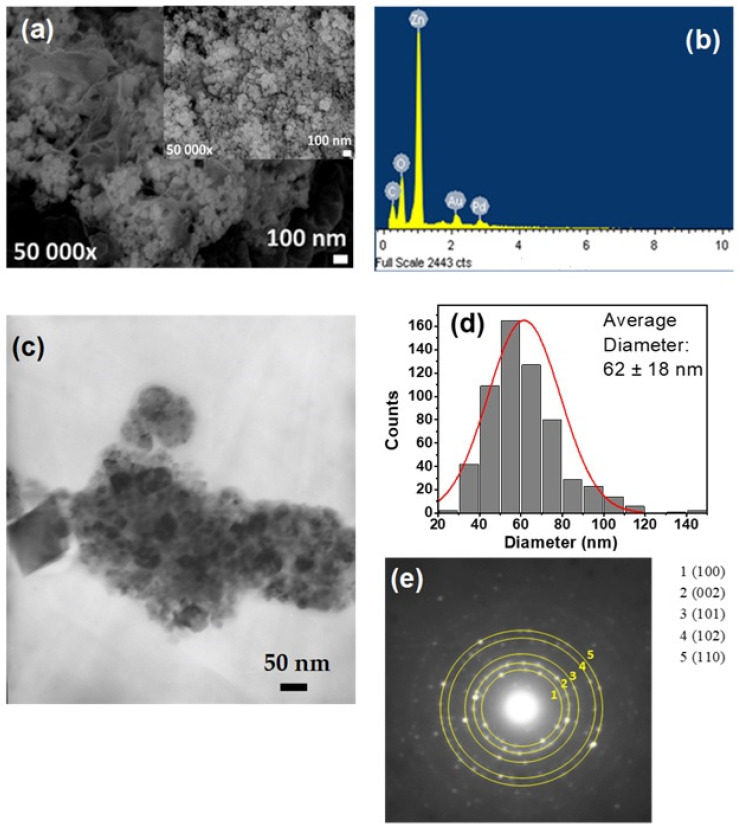
Morphological analysis of the CTS-ZnO compound: (**a**) SEM image of the CTS-ZnO compound at 50,000×, (**b**) EDS diagram confirming the presence of ZnO, (**c**) TEM image a magnification of 50,000×, (**d**) average diameter distribution of inorganic nanoparticles and (**e**) Selected Area Electron Diffraction (SAED) pattern showing the planes corresponding to ZnO structure.

Figure 6a–e shows the evaluation of the morphological and microstructural aspects of the CTS-ZnO composites. The ZnO nanoparticles (ZnO NPs) added to the CTS matrix displayed a semi-spherical morphology (Figure 6a). These particles were not uniformly distributed on the CTS flakes, instead forming agglomerates. The energy-dispersive X-ray spectroscopy (EDS) spectra demonstrate that the metallic oxide particles correspond exclusively to the ZnO NPs (Figure 6b), featuring a particle size distribution from 20 nm and 120 nm, and an average value of 62 ± 18 nm (Figure 6c,d), which is comparable with previous reports [78,79].

### 3.5. Fabrication of PVA-CTS Electrospun Fibers

**Figure 7:**
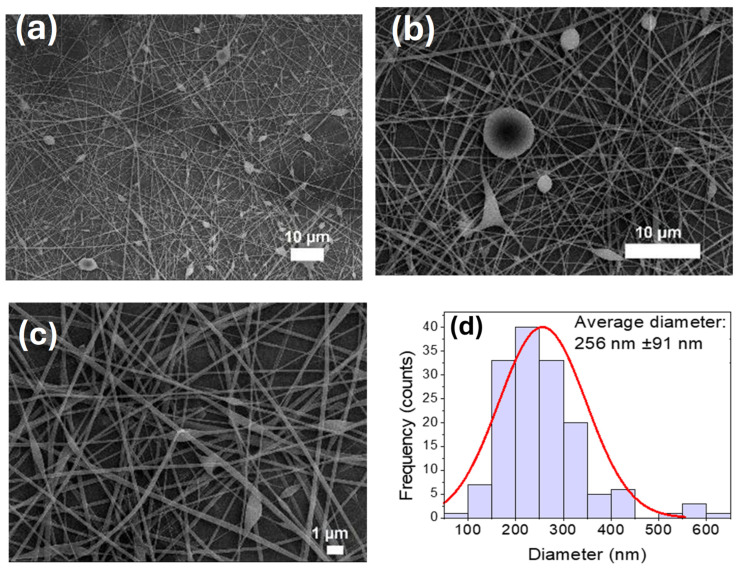
(**a**–**c**) SEM images of the electrospun PVA-CTS fibers at the following conditions: 2 µL/min, 25 cm distance, 22 G gauge needle, 25 kV voltage, 30 cm distance; (**d**) histogram of the average diameters of the electrospun fibers.

The experimental methodology for fabricating scaffolds began with optimizing the production of pure PVA fibers, which served as the main component of the samples. This optimization aimed to achieve efficient fiber collection with minimal defects. Building on prior work, the study focused on 8% PVA electrospun fibers (146–186 kDa), refining the process parameters [59]. Previously, bead-free fibers were obtained using a metallic, cylindrical rotating collector (20 cm diameter, 500 rpm), a flow rate of 3 µL min^−1^, an applied voltage of 25 kV applied voltage, and a 20 cm needle-to-collector distance over 40 h, yielding 11 µm thick fibers. For the current study, pure PVA fiber optimization focused on the collector type, aiming to increase the amount of collected polymer and reduce electrospinning time. Experiments varied the flow rate (1–4 µL min^−1^), applied voltage (15–30 kV), and needle-to-collector distance (15–30 cm). The optimal conditions for 8% pure PVA electrospun fibers with minimal defects were determined to be: a flow rate 2 µL min^−1^, an applied voltage of 25 kV, a distance of 25 cm, and a 22 G needle (0.7 mm inner diameter, 28 mm length). These conditions produced fibers with an average diameter of 501 ± 122 nm, consistent with previous findings for similar PVA concentrations and molecular weights. SEM micrographs and diameter distribution histograms of PVA-CTS can be observed in Figure 7a–d.

The incorporation of chitosan (CTS) into the PVA solution notably decreased the fiber diameter. Pure PVA fibers measured 501 ± 122 nm, while PVA-CTS fibers were significantly smaller at 256 ± 91 nm. This reduction is likely due to the increased electrical conductivity provided by the protonated amino groups (^+^NH_3_) of the acidic CTS. It is well-documented that electrospinning pure CTS is challenging because of its high solution viscosity, mainly due to hydrogen bonding between the NH_2_ and OH^−^ groups of its chains. However, adding PVA helps overcome this hurdle. PVA reduces these hydrogen bridge interactions through inter-polymer chain interactions, which in turn lower the viscosity of the solution and the entanglement threshold of the CTS chains, thereby enabling successful fiber formation via electrospinning [80]. The incorporation of CTS into the PVA solution increased the presence of bead-type defects but decreased the diameter of the electrospun fibers by almost 50%.

### 3.6. Fabrication of PVA-CTS-HA Electrospun Fibers

**Figure 8:**
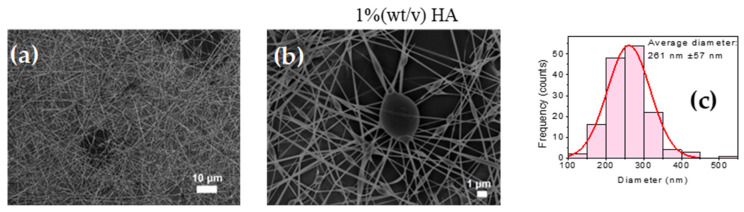

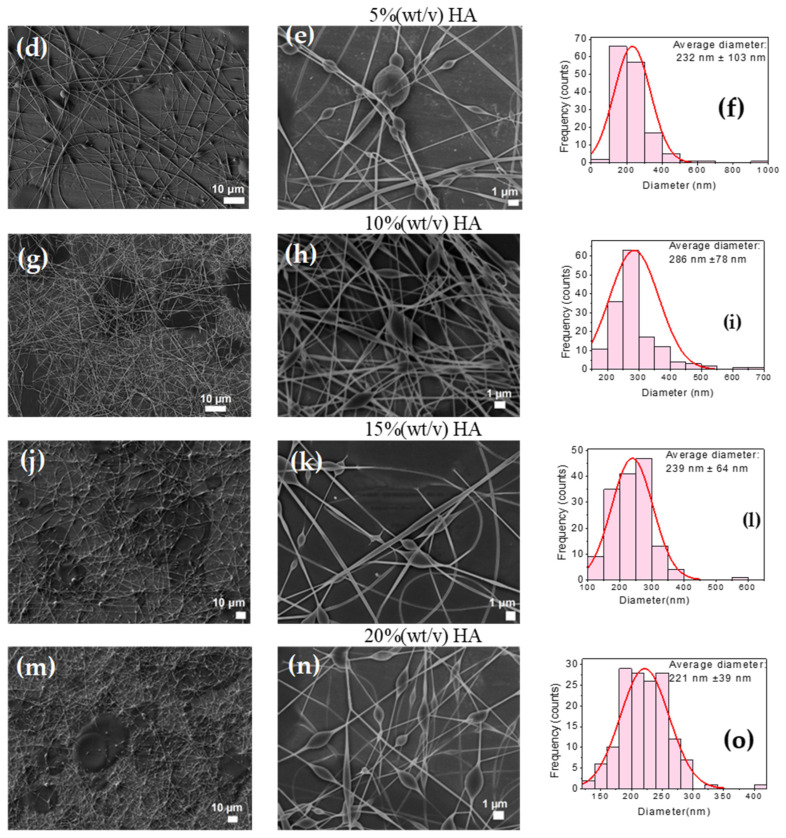
SEM images at different magnifications of the electrospun PVA-QTS-AH fibers at the following conditions: 3 µL/min, 25 cm distance, 22 G gauge needle, 25 kV voltage, 2 µL/min, 30 cm distance; (**a**,**b**) 1% HA, (**d**,**e**) 5% HA, (**g**,**h**) 10% HA, (**j**,**k**) 15% HA, (**l**–**n**) 20% HA; (**c**,**f**,**i**,**l**,**o**) histograms of the average diameters of the electrospun fibers.

To study the effect of HA incorporation on the electrospinning processability of a PVA-CTS blend, fibers were successfully fabricated with HA concentrations of 1, 5, 10, 15, and 20% (*w*/*v*). The optimal electrospinning conditions varied with HA concentration: for 1% HA, a 3 µL/min flow rate, 25 cm distance, and 25 kV voltage were used, while higher concentrations (5–20% HA) required a 2 µL/min flow rate and 30 cm distance. The scanning electron microscopy (SEM) images of these fibers are shown in Figure 8a–o. The figure also shows the histograms of the average fiber diameters corresponding to the respective HA concentrations. The incorporation of different concentrations of HA influenced the fiber morphology, causing the average fiber diameter to change as follows: 261 nm (1% wt/v), 232 nm (5% wt/v), 286 nm (10% wt/v), 239 nm (15% wt/v) and 221 nm (20% wt/v). While the interaction between the protonated amino groups (NH_3_^+^) of CTS and the negative carboxyl groups (COO−) of HA theoretically favors thinner fibers by reducing repulsive charges, no significant decrease in average diameter was consistently observed. Notably, the 1% HA blend produced fibers with the fewest bead-type defects, while higher concentrations resulted in more defects and non-electrospun drops due to increased solution viscosity. Ultimately, the maximum HA content for a bead-free product was determined to be 1%, although fibers with up to 20% HA were successfully produced, demonstrating that its effect on fiber morphology is complex and concentration-dependent.

The incorporation of HA into the PVA-CTS mixture significantly influenced the morphology of the fibers, although no clear trend was observed. The average fiber diameter varied from 221 ± 39 nm to 286 ± 78 nm. The sample with the lowest number of bead-type defects was the mixture with 1% (*w*/*v*) HA. In contrast, samples with 5, 10, 15, and 20% (*w*/*v*) HA showed a higher number of defects, attributed to increase solution viscosity. A sample with 40% (*w*/*v*) HA could not be electrospun because of its excessively high viscosity [51]. The decrease in fiber diameter that occurred with the incorporation of HA is likely due to the interaction between the protonated amino groups (^+^NH_3_) of CTS and the negative carboxyl groups (COO−) of HA. This interaction reduces the repulsive charges of CTS and promotes jet stretching, which results in thinner fibers.

However, a significant decrease in average diameter was not observed. For higher HA concentrations, the fiber diameter remained around 232 ± 103 nm, and bead-type defects and non-electrospun drops were present due to the increased viscosity. In summary, electrospun fibers were successfully obtained from the PVA-CTS-HA blend with a maximum HA content of 20%. The effect of HA appears to be concentration-dependent: at lower concentrations, it helps reduce bead-type defects, but it does not consistently decrease the average fiber diameter. For a completely bead-free result, the maximum HA content in the blend was determined to be 1%.

Briefly, it was observed that incorporating HA into the PVA-CTS mixture significantly improved the morphology of the fibers while decreasing their average diameter, which was ranged from 221 ± 39 to 286 ± 78 nm. Similar results were reported by Hossein et al. [51]. The sample with the lowest number of bead-type defects was the mixture with 1% HA, while the samples with 5, 10, 15 and 20% HA presented a higher number of bead-type defects, attributed to an increase in solution viscosity caused by HA. The sample with 40% HA was not obtained by electrospinning, due to the high viscosity of the solution. The decrease in diameter of the electrospun PVA-CTS-AH fibers due to the incorporation of HA in the blend can be attributed to the interaction between the protonated amino groups (^+^NH_3_) of CTS and the negative carboxyl groups (COOH-) of HA, which decreases the repulsive charges of CTS and favors the jet stretching process, producing thinner fibers. A decrease in average diameter of almost 50% compared to PVA-CTS fibers was not observed, although there was a significant decrease in bead-type defects and a lower narrower size distribution.

The fiber diameter remained largely unchanged, which had an average of 232 ± 261 nm; however, the fibers did present bead-type defects and non-electrospun drops due to the increase in the viscosity of the solution. Overall, it can be concluded that electrospun fibers were successfully obtained from the PVA-CTS-HA blend with a maximum HA content of 20%. The effect of HA on the processing of PVA-CTS solutions by electrospinning favors the formation of fibers with fewer bead-type defects and has no significant influence on the average diameter of the fibers. However, to obtain electrospun fibers without beads the maximum HA content in the blend should be 1%. The main results of the biological evaluation of the PVA-CTS-HA-Ag-ZnO scaffolds at different weight ratios are presented below.

### 3.7. Thickness and Chemical Characterization of Electrospun Fibers

**Figure 9:**
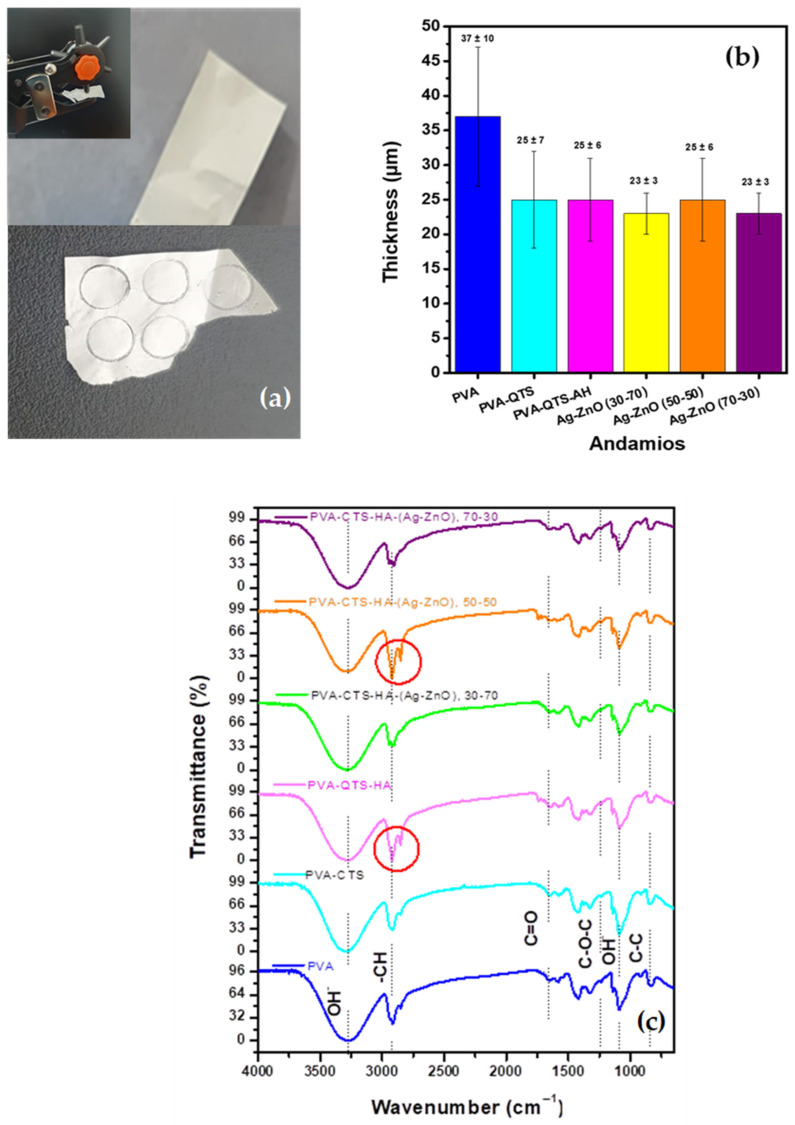
(**a**) Appearance of the electrospun fiber scaffolds on and detached from the aluminum foil substrate; (**b**) average thickness and (**c**) FTIR spectra of the electrospun fiber scaffolds.

Fibrous scaffolds were successfully fabricated by electrospinning solutions containing a blend of polyvinyl alcohol (PVA), chitosan (CTS), and hyaluronic acid (HA), with varying ratios of Ag-ZnO nanoparticles added (30:70, 50:50, and 70:30). The processing time required to produce these scaffolds was 30 h. For comprehensive comparison, the study also included a series of scaffolds with sequential addition of components: pure PVA, followed by PVA-CTS, and finally PVA-CTS-HA. A visual representation of the resulting scaffolds is shown in Figure 9a. Each film exhibited a white color, which remained consistent even after being detached from the aluminum foil substrate.

The incorporation of nanoparticles can affect the physical characteristics of electrospun fibers. Generally, research on electrospun scaffolds for biomedical or biotechnological applications focuses on measuring their chemical and biological properties and does not explain the changes in the physical properties of the fibers [81]. Nanoparticles incorporated into a polymer matrix form nanocomposites. The incorporation of nanoparticles influences some physical properties such as electrical conductivity, which is an electrospinning process parameter that affects fiber diameter. In turn, the morphology and diameter of the nanofibers directly influence the physical properties of the scaffold, such as electrical conductivity, mechanical properties, and thermal stability. The incorporation of inorganic particles is often beneficial, but it requires effective dispersion of the nanoparticles in the matrix, good adhesion at the nanoparticle–polymer interface, and the specific polymer system [82].

The average thickness of the composite electrospun fiber scaffolds was approximately 25 µm. The only exception was the pure PVA scaffold, which had an average thickness of 37 ± 10 µm and required a longer collection time of about 60 h (Figure 9b). This consistency in thickness among the composite samples highlights the reproducibility of the synthesis method. While the ideal thickness for tissue engineering scaffolds is often cited as being in the range of 200–250 µm to support cell infiltration and proliferation, the electrospinning technique allows for the production of much thinner scaffolds [83,84]. This is a crucial point, as the thinner fibrous architecture can be advantageous for several reasons. A reduced thickness can modulate the mechanical properties and facilitate better interaction between the scaffolds’ components and cells, as it mimics the structure of the native extracellular matrix (ECM) more closely [85]. Furthermore, producing thicker scaffolds can be challenging. As noted in the literature, thicknesses above 100 µm can lead to an accumulation of static charges during the electrospinning process, which hinders the uniform deposition of subsequent fibers and limits the production of thicker structures [85]. Therefore, the average thickness of 25 µm is consistent with similar chitosan/polyvinyl alcohol electrospun fibers used for wound healing applications [86]. This thickness is well-suited for applications like wound dressings, where a thin, flexible, and highly porous material is required to facilitate oxygen exchange and moisture management while providing a physical barrier for protection and promoting cellular migration.

Fourier-transform infrared spectroscopy (FTIR) was used to analyze the electrospun fiber scaffolds and identify structural changes within the polymers after incorporating Ag and ZnO nanoparticles. As shown in Figure 9c, the spectra of the pure and composite polymer scaffolds were analyzed.

The spectra of the pure PVA, PVA-CTS, and PVA-CTS-HA scaffolds were quite similar, showing several characteristic vibrational bands consistent with existing literature. For example, the broad peak at 3276 cm^−1^ indicated the stretching vibration of the O-H group, pointing to strong hydrogen bonding. The asymmetric stretching vibration of the C-H group was observed at 2921 cm^−1^, while the stretching of the carbonyl group (C=O) appeared at 1656 cm^−1^. Furthermore, bending vibrations of the -CH_2_ group were detected at 1422 cm^−1^ and 1323 cm^−1^. Other key peaks included the stretching of the glycosidic bond at 1235 cm^−1^, the C-O stretching at 1145 cm^−1^, and the C-C stretching of the acetyl group at 1091 cm^−1^. An in-plane O-H bending vibration was noted at 920 cm^−1^, and a band related to C-C resonance was present at 841 cm^−1^ [87,88]. These bands serve as a clear fingerprint for the PVA polymer, and their presence with slight modifications in the composite spectra confirms the successful blending of the polymers.

The FTIR spectra of the composite scaffolds containing Ag-ZnO nanoparticles were remarkably similar to those of the pure polymer scaffolds, with no significant changes in the overall shape or intensity of the bands. This indicates that the fundamental chemical structure of the polymers was not altered by the nanoparticles. However, one notable difference was a decrease in the intensity of the -CH_2_ stretching vibration at 2921 cm^−1^ in the composite samples. This specific change suggests a physical interaction between the abundant -CH_2_ groups in the PVA backbone and the surface of the Ag and ZnO nanoparticles. This interaction likely influences the vibrational modes of the polymer chains, confirming the successful incorporation and binding of the nanoparticles within the polymer matrix.

Fourier-transform infrared spectroscopy (FTIR) was used to analyze the electrospun fiber scaffolds and to identify structural changes within the polymers after incorporating Ag and ZnO nanoparticles. As shown in Figure 9c, the spectra of the pure and composite polymer scaffolds were analyzed.

### 3.8. In Vitro Analysis of Electrospun Fiber Scaffolds: Determination of Hemolysis Percentage and Cell Viability

**Figure 10:**
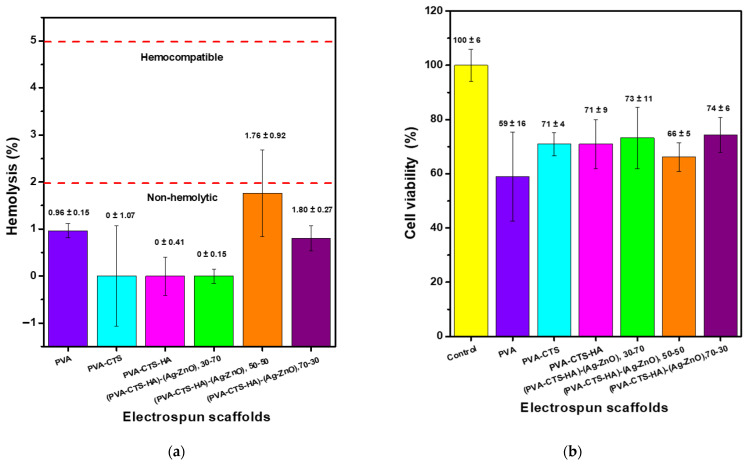
In vitro analysis of PVA-CTS-HA-Ag/ZnO electrospun fiber scaffolds at different weight ratios, (**a**) hemolysis percentage plot and (**b**) cell viability assay.

Figure 10a shows that all the electrospun scaffolds presented a percentage of hemolysis lower than 5%, which, according to the international standard ISO 10993-4, is an acceptable value to consider these materials as hemocompatible. Moreover, the hemolysis percentage of all the materials was below 2%, which indicates that the materials are not only hemocompatible but also non-hemolytic [21]. Additionally, the international standard ISO 10993-5 states that a material can be considered non-cytotoxic when its cell viability is equal to or greater than 70%. As shown in Figure 10b, viability assays revealed that pure PVA electrospun fiber scaffolds were cytotoxic (59 ± 16% viability) to human dermal fibroblasts. In contrast, the incorporation of CTS significantly increased cell viability to non-cytotoxic levels (71 ± 4%), suggesting a remarkable ability of CTS to mitigate the inherent cytotoxicity of PVA. On the other hand, the addition of HA to the PVA-CTS mixture did not produce a further significant increase in cell viability (71 ± 9%), indicating that the non-cytotoxic effect is mainly attributed to CTS.

Regarding the scaffolds containing nanoparticles, it was observed that the Ag-ZnO ratio had a determining impact on cytocompatibility. The sample with a 50-50 ratio (Ag-ZnO) showed reduced viability (66 ± 5%), falling below the non-cytotoxicity threshold, while the samples with ratios of 30-70 and 70-30 showed non-cytotoxic viability and were very similar to each other (73 ± 11% and 74 ± 6%, respectively), being slightly higher for the sample with a higher Ag NPs content. These results suggest that the synergistic effect of Ag-ZnO in the same weight ratios is to increase the cytotoxicity of the scaffold. This may be because, under balanced weight conditions, it can increase the release of Ag^+^ or Zn^2+^ ions, while a higher Ag NPs content promotes slightly greater biocompatibility.

Then, the materials do not interfere with the fibroblast cell cycle, making them suitable for skin cell regeneration and offering broad potential for use as dressing materials in the treatment of chronic wounds, such as diabetic foot ulcers.

## 4. Conclusions

Based on a comprehensive analysis, this study successfully synthesized and characterized hybrid scaffolds combining a polymer blend of polyvinyl alcohol (PVA), chitosan (CTS), and hyaluronic acid (HA) with Ag-ZnO nanoparticles. Using various spectroscopic and microscopic techniques, the researchers confirmed the successful formation and integration of these composite materials. The most important findings are summarized as follows:

The evaluation of HA content in the PVA-CTS-HA mixture suggests that while HA can be added up to 20 wt%, the most uniform morphology and fewest bead-like defects were observed in the mixture with just 1 wt% HA. This is because lower quantities of HA promote the reduction in electrostatic repulsion between the protonated amino groups of CTS and the negative carboxyl groups of HA, resulting in a more stable structure.

The addition of inorganic particles, such as Ag-ZnO nanoparticles, to PVA-CTS-HA scaffolds significantly influenced their structural, morphological, and biological properties. This incorporation is not a simple physical mixture; strong evidence from techniques like FTIR spectroscopy supports the formation of physical interactions between the nanoparticles and the polymer matrix.

Electrospinning proved to be an effective method for fabricating thin, fibrous PVA-CTS-HA-Ag-ZnO scaffolds with an average thickness of 25 µm. This method favored a uniform dispersion of nanoparticles within the polymer matrix, helping to create a more uniform, fibrous structure, which is crucial for mimicking the native extracellular matrix.

These as-prepared scaffolds demonstrated adequate biocompatibility and hemocompatibility; biological evaluation showed that all fabricated scaffolds were hemocompatible, with a hemolysis percentage below 2%, and non-toxic to human dermal fibroblasts, except for the 50-50 Ag-ZnO sample. Thus, these findings confirm that the synthesized electrospun scaffolds are a promising material for biomedical applications. The successful integration of Ag-ZnO nanoparticles into the polymer matrix not only produced a thin, fibrous structure that mimics the native extracellular matrix but also ensures the safety of the materials and their biocompatibility. This research validates the potential of these composite scaffolds as wound dressings, particularly for chronic wounds, given their non-hemolytic and non-toxic properties that support fibroblast cell viability and skin regeneration. To fully confirm this performance, future research should include evaluations of in vitro degradation, cell adhesion tests, contact angle measurements, and tests to determine the mechanical properties.

## Figures and Tables

**Table 1:** Processing conditions for producing electrospun fibers and weight ratios for the preparation of composite scaffolds.

Parameter	Range	CTS + NPs 2 wt %		PVA Solution 8 wt %		HA Solution 2 wt %		Ag-ZnO Nanoparticles in Situ CTS			
								Ag NPs Solution 2 wt %		ZnO NPs Solution 2 wt %	
		%	g	%	g	%	g	%	g	%	g
Needle gauge	20–22 G	39.00	1.95	60.00	3.00	1.00	0.05	30	0.585	70	1.365
Voltage	15, 20, 25 and 30 kV							50	0.975	50	0.975
Flow rate	1, 2, 3 and 4 µL/min							70	1.365	30	0.585
Distance	15, 20, 25 and 30 cm										

## Data Availability

The original contributions presented in this study are included in the article. Further inquiries can be directed to the corresponding authors.

## References

[1] Bhat S.Z., Al-Hajri N., Kanbour S., Ahmadzada M., Borovoy A., Abusamaan M.S., Canner J.K., Nass C., Sherman R.L., Hines K.F. (2024). Glycemic management in diabetic foot ulcers: A comparative analysis of wound and wound-free periods in adults with type 1 and type 2 diabetes. Can. J. Diabetes.

[2] Armstrong D.G., Boulton A.J.M., Bus S.A. (2017). Diabetic foot ulcers and their recurrence. N. Engl. J. Med..

[3] Apelqvist J., Bakker K., van Houtum W.H., Schaper N.C. (2008). Practical guidelines on the management and prevention of the diabetic foot: Based upon the international consensus on the diabetic foot (2007) prepared by the international working group on the diabetic foot. Diabetes Metab. Res. Rev..

[4] Wang G., Jiang Y.-b., Liu Z.-b., Li M.-h., Niu W.-j., Lei Z.-c., Wang B.-w., Lu D.-y., Zhu Y.-w. (2025). Benefits of liquid dressings in post-operative wound dressing of diabetic foot ulcer. Curr. Probl. Surg..

[5] Brouwer R.J., van Reijen N.S., Dijkgraaf M.G., Hoencamp R., Koelemay M.J., van Hulst R.A., Ubbink D.T. (2024). Economic analysis of hyperbaric oxygen therapy for the treatment of ischaemic diabetic foot ulcers. Diving Hyperb. Med..

[6] Castellani L., Arruda S. (2024). Hooked on healing—Fish skin grafts for diabetic foot ulcers. NEJM Evid..

[7] Hinchliffe R.J., Andros G., Apelqvist J., Bakker K., Friederichs S., Lammer J., Lepantalo M., Mills J.L., Reekers J., Shearman C.P. (2012). A systematic review of the effectiveness of revascularization of the ulcerated foot in patients with diabetes and peripheral arterial disease. Diabetes Metab. Res. Rev..

[8] Clark R.A.F. (1985). Cutaneous tissue repair: Basic biologic considerations. I. J. Am. Acad. Dermatol..

[9] Armstrong D.G., Swerdlow M.A., Armstrong A.A., Conte M.S., Padula W.V., Bus S.A. (2020). Five year mortality and direct costs of care for people with diabetic foot complications are comparable to cancer. J. Foot Ankle Res..

[10] Biondo M., Tomasello L., Giordano C., Arnaldi G., Pizzolanti G. (2024). The promising approach of 3D bioprinting for diabetic foot ulcer treatment: A concise review of recent developments. Heliyon.

[11] Paiva M.D.O., Rojas S.D.N. (2016). Pie diabético: ¿podemos prevenirlo?. Rev. Méd. Clín. Condes.

[12] Burgess J.L., Wyant W.A., Abdo Abujamra B., Kirsner R.S., Jozic I. (2021). Diabetic wound-healing science. Medicina.

[13] Wang L., Zhang L., Wu J., Bian Y., Cai L., Hu G., Zhou X. (2025). A comprehensive review of the pathophysiology and management of amniotic fluid embolism. Clin. Exp. Obstet. Gynecol..

[14] Raja J., Maturana M., Kayali S., Khouzam A., Efeovbokhan N. (2023). Diabetic foot ulcer: A comprehensive review of pathophysiology and management modalities. World J. Clin. Cases.

[15] Beckman J., Creager M., Libby P. (2002). Diabetes and atherosclerosis. JAMA J. Am. Med. Assoc..

[16] Park S. (2022). Building vs. Rebuilding epidermis: Comparison embryonic development and adult wound repair. Front. Cell Dev. Biol..

[17] Brings S., Fleming T., Freichel M., Muckenthaler M., Herzig S., Nawroth P. (2017). Dicarbonyls and advanced glycation end-products in the development of diabetic complications and targets for intervention. Int. J. Mol. Sci..

[18] Dawi J., Tumanyan K., Tomas K., Misakyan Y., Gargaloyan A., Gonzalez E., Hammi M., Tomas S., Venketaraman V. (2025). Diabetic foot ulcers: Pathophysiology, immune dysregulation, and emerging therapeutic strategies. Biomedicines.

[19] Khorasani M.T., Joorabloo A., Moghaddam A., Shamsi H., MansooriMoghadam Z. (2018). Incorporation of ZnO nanoparticles into heparinised polyvinyl alcohol/chitosan hydrogels for wound dressing application. Int. J. Biol. Macromol..

[20] Boateng J.S., Matthews K.H., Stevens H.N.E., Eccleston G.M. (2008). Wound healing dressings and drug delivery systems: A review. J. Pharm. Sci..

[21] Sheikholeslami S.A., Esmaeili J., Jalise S.Z., Barati A. (2024). A response surface methodology study on the development of ph-sensitive wound dressings using rhodamine b-loaded chitosan nanoparticles and sodium alginate-based films. Heliyon.

[22] Koupai A.A., Varshosaz J., Tavakoli M., Mirhaj M., Salehi S., Dobakhti F. (2025). Multifunctional tri-layer wound dressing containing zno nanoparticles and igf-1 as an efficient biomaterial for healing of full thickness skin injuries. Asian J. Pharm. Sci..

[23] Wu Z., Lu D., Sun S., Cai M., Lin L., Zhu M. (2025). Material design, fabrication strategies, and the development of multifunctional hydrogel composites dressings for skin wound management. Biomacromolecules.

[24] Liu M., Chen Y., Zhang Y., Zhuang P., Wang J. (2025). Breathable functional aerogel dressings facilitate the healing of diabetic wounds. Biomed. Technol..

[25] Wang L., Wang H., Dang H., Niu B., Yan H., Guo R., Wang H., Zhou P. (2025). An adhesive, antibacterial hydrogel wound dressing fabricated by dopamine-grafted oxidized sodium alginate and methacrylated carboxymethyl chitosan incorporated with Cu(II) complex. Biomater. Adv..

[26] Xie X., Chen Y., Wang X., Xu X., Shen Y., Khan A.u.R., Aldalbahi A., Fetz A.E., Bowlin G.L., El-Newehy M. (2020). Electrospinning nanofiber scaffolds for soft and hard tissue regeneration. J. Mater. Sci. Technol..

[27] Shakil U.A., Abu Hassan S.B., Yahya M.Y., Rejab M.R.M. (2022). A focused review of short electrospun nanofiber preparation techniques for composite reinforcement. Nanotechnol. Rev..

[28] Liu Y., Zhou S., Gao Y., Zhai Y. (2019). Electrospun nanofibers as a wound dressing for treating diabetic foot ulcer. Asian J. Pharm. Sci..

[29] Li W., He J., Chen Q., Bao F., Huo Y., Deng J., Lin Q., Luo F. (2025). Enhancement of oryzanol application by constructing modified β-cd inclusion complex and polycaprolactone-chitosan electrospun fiber membranes: Perspectives on wound dressings and grape preservation. Food Chem..

[30] Madani M., Cruz C.D., Gounani Z., Baniasadi H., Tammela P., Laaksonen T., Niskanen J., Seppälä J. (2025). Functionalized cellulose nanocrystals reinforced pla-gelatin electrospun fibers for potential antibacterial wound dressing and coating applications. Int. J. Biol. Macromol..

[31] Breitenbach G.L., Caldas B.S., Pellá M.C.G., Muniz E.C., Dragunski D.C. (2024). Graphene oxide (go)-reinforced curcumin (cur)-loaded zein-based electrospun nanofibers for potential wound dressing purposes. Colloids Surf. A Physicochem. Eng. Asp..

[32] Pirzadeh K., Torkian L., Asli M.D. (2024). Coaxial electrospun wound dressing integrated with ag-doped hydroxyapatite for wound healing: Tetracycline delivery. Mater. Today Commun..

[33] Gruppuso M., Turco G., Marsich E., Porrelli D. (2023). Antibacterial and bioactive multilayer electrospun wound dressings based on hyaluronic acid and lactose-modified chitosan. Biomater. Adv..

[34] Marjani M.E., Hmtshirazi R., Mohammadi T. (2024). CDI crosslinked chitosan/poly (vinyl alcohol) electrospun nanofibers loaded with achillea millefolium and viola extract: A promising wound dressing. Carbohydr. Polym..

[35] Zou P., Lee W.-H., Gao Z., Qin D., Wang Y., Liu J., Sun T., Gao Y. (2020). Wound dressing from polyvinyl alcohol/chitosan electrospun fiber membrane loaded with oh-cath30 nanoparticles. Carbohydr. Polym..

[36] Alizadeh S., Nassiri M., Farahmandian N., Farshi P., Aliakbar Ahovan Z., Majidi J., Hashemi A., Shafikhani S.H., Moroni L., Gholipourmalekabadi M. (2024). Engineering of a bilayer antibacterial wound dressing from bovine pericardium and electrospun chitosan/PVA/antibiotics for infectious skin wounds management: An in vitro and in vivo study. Int. J. Biol. Macromol..

[37] Dong Z., Cui H., Wang Y., Wang C., Li Y., Wang C. (2020). Biocompatible AIE material from natural resources: Chitosan and its multifunctional applications. Carbohydr. Polym..

[38] Moeini A., Pedram P., Makvandi P., Malinconico M., Gomez d’Ayala G. (2020). Wound healing and antimicrobial effect of active secondary metabolites in chitosan-based wound dressings: A review. Carbohydr. Polym..

[39] Wang M., Roy A.K., Webster T.J. (2017). Development of chitosan/poly(vinyl alcohol) electrospun nanofibers for infection related wound healing. Front. Physiol..

[40] Zhang W., Khan A., Ezati P., Priyadarshi R., Sani M.A., Rathod N.B., Goksen G., Rhim J.-W. (2024). Advances in sustainable food packaging applications of chitosan/polyvinyl alcohol blend films. Food Chem..

[41] Chen Y., Etxabide A., Seyfoddin A., Ramezani M. (2023). Fabrication and characterisation of poly(vinyl alcohol)/chitosan scaffolds for tissue engineering applications. Mater. Today Proc..

[42] Fathi A., Khanmohammadi M., Goodarzi A., Foroutani L., Mobarakeh Z.T., Saremi J., Arabpour Z., Ai J. (2020). Fabrication of chitosan-polyvinyl alcohol and silk electrospun fiber seeded with differentiated keratinocyte for skin tissue regeneration in animal wound model. J. Biol. Eng..

[43] Talebi M., Ghale R.A., Asl R.M., Tabandeh F. (2025). Advancements in characterization and preclinical applications of hyaluronic acid-based biomaterials for wound healing: A review. Carbohydr. Polym. Technol. Appl..

[44] Séon-Lutz M., Couffin A.-C., Vignoud S., Schlatter G., Hébraud A. (2019). Electrospinning in water and in situ crosslinking of hyaluronic acid/cyclodextrin nanofibers: Towards wound dressing with controlled drug release. Carbohydr. Polym..

[45] Su X., Geng X., Zhang Y., Shi Y., Zhao L. (2024). Microenvironmental pH modulating oxygen self-boosting microalgal prodrug carboxymethyl chitosan/hyaluronic acid/puerarin hydrogel for accelerating wound healing in diabetic rats. Int. J. Biol. Macromol..

[46] Blinov A., Rekhman Z., Yasnaya M., Gvozdenko A., Golik A., Kravtsov A., Shevchenko I., Askerova A., Prasolova A., Pirogov M. (2025). Enhancement of stability and activity of zinc carbonate nanoparticles using chitosan, hydroxyethyl cellulose, methyl cellulose and hyaluronic acid for multifaceted applications in medicine. Int. J. Biol. Macromol..

[47] Sayyar Z., Hosseini Z., Beheshtizadeh N. (2024). Developing curcumin loaded-magnetic montmorillonite nanoparticles/polyvinyl alcohol/hyaluronic acid/chitosan nanofiber mats as a wound dressing. J. Drug Deliv. Sci. Technol..

[48] Bozdag M., Urek F., Cesur S., Sahin A., Gunduz O. (2025). Bovine serum albumin (bsa)-loaded polyvinyl alcohol (pva)/chitosan (ch)/hydroxyapatite (ha) electrospun nanofibers for bone tissue regeneration. J. Drug Deliv. Sci. Technol..

[49] Salim S.A., Taha A.A., Khozemy E.E., El-Moslamy S.H., Kamoun E.A. (2022). Electrospun zinc-based metal organic framework loaded-pva/chitosan/hyaluronic acid interfaces in antimicrobial composite nanofibers scaffold for bone regeneration applications. J. Drug Deliv. Sci. Technol..

[50] Alkabli J. (2024). Recent advances in the development of chitosan/hyaluronic acid-based hybrid materials for skin protection, regeneration, and healing: A review. Int. J. Biol. Macromol..

[51] Hosseini H., Shahraky M.K., Amani A., Landi F.S. (2021). Electrospinning of polyvinyl alcohol/chitosan/hyaluronic acid nanofiber containing growth hormone and its release investigations. Polym. Adv. Technol..

[52] Mohamady Hussein M.A., Guler E., Rayaman E., Cam M.E., Sahin A., Grinholc M., Sezgin Mansuroglu D., Sahin Y.M., Gunduz O., Muhammed M. (2021). Dual-drug delivery of ag-chitosan nanoparticles and phenytoin via core-shell pva/pcl electrospun nanofibers. Carbohydr. Polym..

[53] Rehan Ansari M., Agrohi P., Rao Peta K. (2024). Effect of citric acid on structural, optical, morphological properties of ZnO and the bactericidal applications against human pathogenic bacteria *E. coli* DH5α. Mater. Today Proc..

[54] Cao S., Li Q., Zhang S., Liu Z., Lv X., Chen J. (2022). Preparation of biodegradable carboxymethyl cellulose/dopamine/Ag nps cryogel for rapid hemostasis and bacteria-infected wound repair. Int. J. Biol. Macromol..

[55] Park S.-j., Nisar S.S., Choe H.-C. (2025). Enhanced surface of collagen-Zn-Ag-Ha coated ti-6al-4v alloy for biocompatibility. Surf. Interfaces.

[56] Li X., Pang L., Duan J., Huang N., Chen X., Huang W., Liu Y., Fu C., Zhang C., Tu H. (2025). Eco-friendly antibacterial electrospinning nanofibrous film containing nano-silver green-synthesized by natural glycoprotein for infected wound healing. J. Colloid Interface Sci..

[57] Abdelgawad A.M., Hudson S.M., Rojas O.J. (2014). Antimicrobial wound dressing nanofiber mats from multicomponent (chitosan/silver-nps/polyvinyl alcohol) systems. Carbohydr. Polym..

[58] Mujeeb Rahman P., Muraleedaran K., Mujeeb V.M.A. (2015). Applications of chitosan powder with in situ synthesized nano ZnO particles as an antimicrobial agent. Int. J. Biol. Macromol..

[59] Santiago-Castillo K., Del Angel-López D., Torres-Huerta A.M., Domínguez-Crespo M.A., Palma-Ramírez D., Willcock H., Brachetti-Sibaja S.B. (2021). Effect on the processability, structure and mechanical properties of highly dispersed in situ Zno:Cs nanoparticles into pva electrospun fibers. J. Mater. Res. Technol..

[60] Panda P.K., Dash P., Yang J.-M., Chang Y.-H. (2022). Development of chitosan, graphene oxide, and cerium oxide composite blended films: Structural, physical, and functional properties. Cellulose.

[61] Wang X., Tang R., Zhang Y., Yu Z., Qi C. (2016). Preparation of a novel chitosan based biopolymer dye and application in wood dyeing. Polymers.

[62] Panda P.K., Yang J.-M., Chang Y.-H., Su W.-W. (2019). Modification of different molecular weights of chitosan by p-coumaric acid: Preparation, characterization and effect of molecular weight on its water solubility and antioxidant property. Int. J. Biol. Macromol..

[63] Branca C., D’Angelo G., Crupi C., Khouzami K., Rifici S., Ruello G., Wanderlingh U. (2016). Role of the OH and NH vibrational groups in polysaccharide-nanocomposite interactions: A ftir-atr study on chitosan and chitosan/clay films. Polymer.

[64] Berthomieu C., Hienerwadel R. (2009). Fourier transform infrared (ftir) spectroscopy. Photosynth. Res..

[65] Nie B., Stutzman J., Xie A. (2005). A vibrational spectral maker for probing the hydrogen-bonding status of protonated asp and glu residues. Biophys. J..

[66] Fernandes Queiroz M., Melo K.R., Sabry D.A., Sassaki G.L., Rocha H.A. (2015). Does the use of chitosan contribute to oxalate kidney stone formation?. Mar. Drugs.

[67] Hou W., Cronin S.B. (2013). A review of surface plasmon resonance-enhanced photocatalysis. Adv. Funct. Mater..

[68] Ansari M.A., Ali K., Farooqui Z., Al-Dossary H.A., Zubair M., Musarrat J. (2021). Nanotechnology and diabetic foot ulcer: Future prospects. Diabetic Foot Ulcer: An Update.

[69] Paramelle D., Sadovoy A., Gorelik S., Free P., Hobley J., Fernig D.G. (2014). A rapid method to estimate the concentration of citrate capped silver nanoparticles from uv-visible light spectra. Analyst.

[70] Sivakumar A., Rita A., Sahaya Jude Dhas S., Martin Britto Dhas S.A. (2020). Tuning of surface plasmon resonance of silver nano particles by shock waves for plasmonic device applications. Opt. Laser Technol..

[71] Huang Q., Jiao Z., Li M., Qiu D., Liu K., Shi H. (2013). Preparation, characterization, antifungal activity, and mechanism of chitosan/TiO_2_ hybrid film against bipolaris maydis. J. Appl. Polym. Sci..

[72] Davis K., Yarbrough R., Froeschle M., White J., Rathnayake H. (2019). Band gap engineered zinc oxide nanostructures via a sol–gel synthesis of solvent driven shape-controlled crystal growth. RSC Adv..

[73] Ocakoglu K., Mansour S.A., Yildirimcan S., Al-Ghamdi A.A., El-Tantawy F., Yakuphanoglu F. (2015). Microwave-assisted hydrothermal synthesis and characterization of ZnO nanorods. Spectrochim. Acta Part A Mol. Biomol. Spectrosc..

[74] Jang M.-K., Kong B.-G., Jeong Y.-I., Lee C.H., Nah J.-W. (2004). Physicochemical characterization of α-chitin, β-chitin, and γ-chitin separated from natural resources. J. Polym. Sci. Part A Polym. Chem..

[75] Jampafuang Y., Tongta A., Waiprib Y. (2019). Impact of crystalline structural differences between α- and β-chitosan on their nanoparticle formation via ionic gelation and superoxide radical scavenging activities. Polymers.

[76] Banica R., Ursu D., Svera P., Sarvas C., Rus S.F., Novaconi S., Kellenberger A., Racu A.V., Nyari T., Vaszilcsin N. (2016). Electrical properties optimization of silver nanowires supported on polyethylene terephthalate. Part Sci. Technol..

[77] Badawy M.E.I., Lotfy T.M.R., Shawir S.M.S. (2019). Preparation and antibacterial activity of chitosan-silver nanoparticles for application in preservation of minced meat. Bull. Natl. Res. Cent..

[78] Asgari-Targhi G., Iranbakhsh A., Oraghi Ardebili Z., Hatami Tooski A. (2021). Synthesis and characterization of chitosan encapsulated zinc oxide (ZnO) nanocomposite and its biological assessment in pepper (*Capsicum annuum*) as an elicitor for in vitro tissue culture applications. Int. J. Biol. Macromol..

[79] Ahmad Yusof N.A., Mat Zain N., Pauzi N. (2019). Synthesis of chitosan/zinc oxide nanoparticles stabilized by chitosan via microwave heating. Bull. Chem. React. Eng. Catal..

[80] Mata G.C., Morais M.S., Oliveira W.P., Aguiar M.L. (2022). Composition effects on the morphology of pva/chitosan electrospun nanofibers. Polymers.

[81] Langwald S., Ehrmann A., Sabantina L. (2023). Measuring physical properties of electrospun nanofiber mats for different biomedical applications. Membranes.

[82] Kausar A. (2020). Polymeric nanocomposite via electrospinning: Assessment of morphology, physical properties and applications. J. Plast. Film Sheeting.

[83] Telemeco T.A., Ayres C., Bowlin G.L., Wnek G.E., Boland E.D., Cohen N., Baumgarten C.M., Mathews J., Simpson D.G. (2005). Regulation of cellular infiltration into tissue engineering scaffolds composed of submicron diameter fibrils produced by electrospinning. Acta Biomater..

[84] Zhang Z., Hu J., Ma P.X. (2012). Nanofiber-based delivery of bioactive agents and stem cells to bone sites. Adv. Drug Deliv. Rev..

[85] Vaquette C., Cooper-White J. (2013). A simple method for fabricating 3-D multilayered composite scaffolds. Acta Biomater..

[86] Charernsriwilaiwat N., Rojanarata T., Ngawhirunpat T., Opanasopit P. (2014). Electrospun chitosan/polyvinyl alcohol nanofibre mats for wound healing. Int. Wound J..

[87] Panda P.K., Yang J.-M., Chang Y.-H. (2021). Water-induced shape memory behavior of poly (vinyl alcohol) and p-coumaric acid-modified water-soluble chitosan blended membrane. Carbohydr. Polym..

[88] Sharma R., Singh N., Gupta A., Tiwari S., Tiwari S.K., Dhakate S.R. (2014). Electrospun chitosan–polyvinyl alcohol composite nanofibers loaded with cerium for efficient removal of arsenic from contaminated water. J. Mater. Chem. A.

